# Health care professionals’ attitudes towards evidence-based medicine in the workers’ compensation setting: a cohort study

**DOI:** 10.1186/s12911-017-0460-2

**Published:** 2017-05-22

**Authors:** Nieke A. Elbers, Robin Chase, Ashley Craig, Lyn Guy, Ian A. Harris, James W. Middleton, Michael K. Nicholas, Trudy Rebbeck, John Walsh, Simon Willcock, Keri Lockwood, Ian D Cameron

**Affiliations:** 1 Sydney Medical School Northern, University of Sydney; Northern Sydney Local Health District, St Leonards, Australia; 2 Tyrrell Consulting, Adelaide, South Australia Australia; 3 School of Health Sciences, University of Newcastle, Newcastle, Australia; 4 Ingham Institute for Applied Medical Research, South Western Sydney Clinical School, UNSW, Sydney, Australia; 5 Discipline of Physiotherapy, Faculty of Health Sciences, University of Sydney, Sydney, Australia; 6 Macquarie University Hospital and Health Sciences Centre, Sydney, Australia

**Keywords:** Evidence-based medicine, Workers’ compensation process, Health care practitioners, Guidelines

## Abstract

**Background:**

Problems may arise during the approval process of treatment after a compensable work injury, which include excess paperwork, delays in approving services, disputes, and allegations of over-servicing. This is perceived as undesirable for injured people, health care professionals and claims managers, and costly to the health care system, compensation system, workplaces and society. Introducing an Evidence Based Medicine (EBM) decision tool in the workers’ compensation system could provide a partial solution, by reducing uncertainty about effective treatment. The aim of this study was to investigate attitudes of health care professionals (HCP) to the potential implementation of an EBM tool in the workers’ compensation setting.

**Methods:**

The study has a mixed methods design. The quantitative study consisted of an online questionnaire asking about self-reported knowledge, attitudes and behaviour to EBM in general. The qualitative study consisted of interviews about an EBM tool being applied in the workers’ compensation process. Participants were health care practitioners from different clinical specialties. They were recruited through the investigators’ clinical networks and the workers’ compensation government regulator’s website.

**Results:**

Participants completing the questionnaire (*n* = 231) indicated they were knowledgeable about the evidence-base in their field, but perceived some difficulties when applying EBM. General practitioners reported having the greatest obstacles to applying EBM. Participants who were interviewed (*n* = 15) perceived that an EBM tool in the workers’ compensation setting could potentially have some advantages, such as reducing inappropriate treatment, or over-servicing, and providing guidance for clinicians. However, participants expressed substantial concerns that the EBM tool would not adequately reflect the impact of psychosocial factors on recovery. They also highlighted a lack of timeliness in decision making and proper assessment, particularly in pain management.

**Conclusions:**

Overall, HCP are supportive of EBM, but have strong concerns about implementation of EBM based decision making in the workers’ compensation setting. The participants felt that an EBM tool should not be applied rigidly and should take into account clinical judgement and patient variability and preferences. In general, the treatment approval process in the workers’ compensation insurance system is a sensitive area, in which the interaction between HCP and claims managers can be improved.

**Electronic supplementary material:**

The online version of this article (doi:10.1186/s12911-017-0460-2) contains supplementary material, which is available to authorized users.

## Background

Timely access to appropriate treatment and support after injury is crucial for optimal outcomes. Appropriate treatment is usually provided through the regular public or private health care system. In case of a compensable injury, for example a work-related or transport injury, injured people may receive treatment funded by the compensation system. In Australia, work injuries are covered and compensated by state regulated workers’ compensation systems. The workers’ compensation system compensates medical and vocational rehabilitation and income support for workers during incapacity. In New South Wales (NSW), the workplace injury management system is called SafeWork, and is mandated by Workers’ Compensation Legislation NSW, Australia [1]. In general, the aim of the system is to provide prompt, effective and proactive management of work-related injuries.

The approval process for a compensable work injury, however, can be onerous and take a significant amount of time, particularly in complex cases. It is burdensome for health care professionals, due to the amount of paperwork required in order to get treatment approved. Claims managers, who often have a high caseload, have to review the request and investigate the adequacy, appropriateness, and effectiveness of the recommended treatment before making a decision. This means that it often takes more time before a treatment can be delivered in comparison to care delivered outside the workers’ compensation setting (for which no approval is needed). The decision-making and approval processes needs thought and reason and is not automated. Disputes can arise when a claims manager denies certain treatments on the basis of a perceived lack of evidence, which may be due to lack of knowledge about the evidence, conflicting evidence, or different interpretation of the evidence. Additionally, some treatments may be provided at a frequency greater than is clinically justified and this may represent over-servicing. Approval of non-evidence based treatments also occurs [2], for reasons outlined above where the claims managers may not be aware of the evidence of harm (such as, collars for whiplash injury), or because they recognise that some treatments are already common practice even though clear evidence is not available (for example, hot/cold packs for low back pain). In summary, delays in treatment approval, disputes, controversial denial of treatment, overtreatment and approval of non-evidence based treatment are undesirable, can be harmful to injured people and are costly to the health care system, compensation system, workplaces and society.

In response to challenges experienced in the treatment approval process, the workers’ compensation scheme in NSW Australia is considering implementing an electronic Evidence Based Medicine (EBM) guideline tool. The tool is a North American tool but it is used worldwide. In general, EBM guideline tools provide an extensive overview of evidence based treatments and guidelines for a condition. Each referenced study is evaluated using a 30-step grading system, including evaluating sample size, conflict of interest, study design, potential bias, and statistical significance (described in the tool’s user manual, accessed March 2016, not publicly available). The summary of evidence includes an evaluation of 1) trade-off between risks versus benefits, 2) magnitude of effect of an intervention, 3) availability of dependable sources of the treatment, 4) education and experience of providers, 5) consistency of study outcomes, and 6) variability of treatment parameters being studied. The conclusion about whether the health care service is recommended or not, is made by a multidisciplinary advisory board convened by the company that has developed the tool. The names and backgrounds of the board members are provided in the tool’s user manual (accessed March 2016, not publicly available). Claims managers can use the summary and conclusion to make a decision about whether or not to (automatically) approve the treatment. In addition to an overview of recommended treatments, the EBM tool also provides an average number of calendar days of return to work (RTW) by tenth percentiles per injury type, based on the average (local, national, or international) claims data.

The workers’ compensation scheme expects that such a tool could reduce the uncertainty about the appropriateness and effectiveness of a treatment. Reducing the uncertainty could speed up the decision-making process and reduce the need to seek second opinions from medical examiners. Health care practitioners would no longer have to complete additional paperwork for those evidence-based treatments. In general, it could facilitate a common understanding across those requesting treatment/services and those reviewing the services requested.

The tool is primarily developed to be used by claims managers. However, the treatment approval process is an interaction between claims managers and health care professionals. Therefore, it is of interest to investigate how NSW health care professionals think about an EBM tool applied to the workers’ compensation setting in NSW, Australia.

Investigating health care practitioners’ opinion about an EBM tool implies investigating perceptions about EBM in general. “Evidence Based Medicine is the conscientious, explicit, and judicious use of current best evidence in making decisions about the care of individual patients. The practice of evidence based medicine means integrating individual clinical expertise with the best available external clinical evidence from systematic research” [3]. EBM is now an accepted part of clinical practice, however, not without polarized standpoints [4, 5]. The supporters claim that adopting an EBM approach includes positive changes in health professional behaviour, improvements in treatments, less variability between health care provided by different practitioners, and potential cost containment. The critics take the position that EBM is “cookbook medicine”, is unable to account for individual patient factors and involves decreased professional freedom [4, 5]. Several barriers to using EBM have been identified, concerning a lack of awareness of and/or familiarity with the evidence, a lack of agreement, reduced self-efficacy, and/or motivation to apply, and the inability to reconcile EBM with patient preferences, or lack of time and resources [6]. Barriers might be perceived differently for different health care specialties. Furthermore, it is not known how health care professionals think about EBM being applied in the workers’ compensation setting, in which another person (that is, a claims manager) is deciding what treatment should be approved.

The aims of the current study are twofold: to explore health care professionals’ attitudes to (1) EBM, in general, and (2) an EBM tool applied in a workers’ compensation setting, specifically. For attitudes to EBM in general, it was investigated whether clinical specialties differ in self-reported knowledge, attitudes, and behaviour to EBM. The attitudes to an EBM tool applied in the workers’ compensation setting were investigated without any pre-set hypotheses or direction other than investigating the advantages and disadvantages.

## Methods

The method used was a cross-sectional mixed methods design. A quantitative study was conducted to examine whether there were differences in attitudes to EBM as a function of clinical specialty, demographics and job characteristics. A qualitative study was also conducted to explore how health care professionals feel about an EBM tool being applied in the workers’ compensation domain. The quantitative and qualitative studies were conducted simultaneously. The Northern Sydney Local Health District Human Research Ethics Committee approved the study protocol.

### Quantitative study

#### Participants

Participants were health care professionals with a background in chiropractic, clinical psychology, general medical practice, injury management, musculoskeletal medicine, occupational medicine, pain medicine, physiotherapy, rehabilitation medicine, rheumatology and orthopaedic surgery. Participants were recruited by the co-authors, all experienced health care practitioners and opinion leaders in the health care professions under investigation. Participants were recruited using the co-authors’ networks and using lists of allied health care professionals on the NSW workers’ compensation government regulator’s website (www.workcover.nsw.gov.au). The proportion of participants approached via the health care professionals’ networks versus the regulator’s website was about 50:50. Recruitment was achieved by sending invitations by email. If there was no email address provided, recruitment was via fax. The email or fax contained an invitation to complete the online questionnaire and the participant information sheet. Recruitment and data collection occurred between December 2015 and March 2016.

#### Questionnaire

The questionnaire began with background information about the gap between evidence and practice and examples of potential barriers from the literature were provided. Evidence based practices were defined as “use [of] peer reviewed publications, or other peer reviewed materials, that provide evidence for the effectiveness for specific treatments”. Participants were asked for the percentage of their clinical practice they felt was evidence based [7], followed by questions about potential barriers towards EBM. The barriers presented were based on items derived from a systematic review [6]﻿, being lack of awareness, lack of familiarity, lack of agreement, lack of self-efficacy, lack of outcome expectancy, lack of motivation, inability to reconcile EBM with patient preferences, lack of time, lack of resources, lack of organisational support, and/or lack of financial reimbursement [6]. These barriers were grouped in three themes: perceived knowledge, attitudes, and behaviour [6]. To simplify interpretation, the barriers were formulated as positive statements. All questions were presented as Likert scales with 5-levels (1 = strongly disagree, 2 = disagree, 3 = neutral, 4 = agree, 5 = strongly agree). The questionnaire is included in the Additional file 1. The original questionnaire contained some additional questions about barriers to using evidence-based guidelines, usage of an electronic guideline tool, and positive and negative keywords associated with EBM. These questions were added by the funder (a government workers’ compensation regulatory agency). For conciseness purposes, the analyses are not discussed in this paper but can be found in the internal report to the workers’ compensation regulatory agency [8]. Finally, the participants were asked to indicate their age, sex, clinical specialty, work experience, work hours, clinical setting and whether they provided services to the workers’ compensation setting. The questionnaire was programmed in Survey Monkey, an online software medium for creating questionnaires (www.surveymonkey.com).

#### Data analysis

For descriptive statistics, means, standard deviations (SD), median, and frequencies were computed for the demographic characteristics, perceived EBM adherence, EBM subscales (perceived knowledge, attitudes and behaviour), and EBM items individually. Differences between the EBM subscales for perceived knowledge, attitudes and behaviour were also explored. Because the data was not normally distributed, non-parametric tests were used.

Kruskal-Wallis (KW) tests were used to explore the differences between clinical specialties and perceived EBM adherence. KW tests were also used to analyse the differences between demographic and job characteristics and perceived EBM adherence. Seven Mann–Whitney (MW) post hoc analyses were conducted to explore which clinical specialties differed from each other with respect to perceived EBM adherence. The significance threshold was *p* < .05.

Some clinical specialties were clustered based on similarity between clinical issues addressed by the specialties. Therefore, musculoskeletal medicine, occupational medicine and rheumatology merged into one category. Pain and rehabilitation medicine were formed into a second category. This means that only 8 categories were included in the analysis. Because post hoc analyses comparing 8 clinical specialties with each other would yield as many as 64 comparisons, MW analyses were conducted only for the lowest scoring specialty: that is, by choosing that specialty as the reference category. A KW analysis was conducted to analyse the differences between clinical specialties to the question whether payment systems can influence decisions about treatment. Finally, it was investigated whether demographic and job characteristics were associated with different perceived EBM adherence (KW analyses).

### Qualitative study

#### Participants

Participants were health care professionals with backgrounds in general practice, orthopaedic surgery, occupational medicine, rehabilitation medicine, pain medicine, physiotherapy, chiropractic, and clinical psychology. The inclusion criterion was that the participant should have had experience with treating patients in the workers’ compensation system. The qualitative study used a grounded theory approach, involving the construction of theory through the analysis of data [9]. Interviews were conducted until data saturation was reached, that is, when no new themes emerge. Participants were recruited through the clinical networks and other resources, available to the authors. Purposeful sampling was applied, meaning that the co-authors recruited participants that they knew to have opinions across the spectrum with reference to EBM principles. Some co-authors, being opinion leaders in the health care specialties under investigation, were also interviewed to capture their view of the issues influencing treatment of injured workers. The potential participants received an email with the participant information sheet. Participants were asked to sign a confidentiality agreement. Recruitment and data collection occurred in January 2016 to March 2016. Participants were offered a $50 shopping voucher as reimbursement for their time.

#### Interviews

The interviews consisted of three parts. Firstly, the participants were asked about their experience with treating patients in the workers’ compensation setting as compared to non- workers’ compensation patients, regarding timeliness of treatment, the number of treatment sessions, treatment content, and the amount of paperwork. Secondly, participants were informed about the two main functionalities of the EBM tool being: 1) the treatment recommendation part, and 2) the return to work part. The treatment recommendation part consisted of an overview (print screen) from the tool, showing some examples of recommended - and non-recommended treatments. The chosen examples were relevant to participant’s medical specialty. Participants were informed that treatments would be coded according to a flag system, which would distinguish between treatments that would be automatically approved (because studies show a good effect or low costs), and treatments that would be reviewed or denied (because studies show only an effect under certain circumstances or because studies show no effect). For example, for low back pain injury, 6 sessions of physiotherapy would be automatically approved, that is, without paperwork, whereas work hardening and artificial disc replacement would be reviewed or denied. For the return-to-work part, we explained that, per condition, the tool would show a summary about how many days it will take for injured people to go back to work, based on claims data. A yellow flag is raised when an injured person is not back at work after the number of days at which 50% of injured people with this injury are back at work. A red flag is raised when the injured person is not back at work when 90% of claims with this injury are back at work. The flags would be an indication for the claims manager that close monitoring is needed. The return-to-work expectancy could be adjusted based on individual circumstances. Participants were asked how they would feel about claims managers adopting such a tool. Thirdly, participants were asked whether they would consider using the tool in their clinical setting. Finally, participants were asked for their demographic and job characteristics. For conciseness purposes, this paper reports only the opinions about the tool in the workers’ compensation setting. The complete findings and interview scheme can be found in the internal report for the workers’ compensation agency [8].

The interview format was discussed with the co-authors and pilot tested twice to measure the duration and to evaluate the content. Based on the test interviews, the examples regarding which treatment would be approved or not were adapted for each clinical specialty rather than having one interview scheme for all clinical specialties. The interviews were conducted by the principal investigator [NE] by telephone. The average duration of the interviews was 45 minutes. Participants provided informed consent and signed a confidentiality agreement before the interview. Interviews were audio recorded and transcribed.

#### Data analysis

Data was analysed using a grounded theory approach, which involves three sequential phases of coding: open, axial and selective coding [9]. In the open coding phase, the investigators identified preliminary concepts based on the themes in the interview scheme. Consistent with a framework approach [10], we applied labels associated with EBM, in general, such as quality of evidence, patient preferences, individual differences and searched for additional labels related to the workers’ compensation setting. In the axial coding process, the labels were restructured, sub-labels were applied and new labels emerged. During the selective coding, all the transcripts were re-analysed based on the refinement that occurred during axial coding. The interviews were analysed in duplicate by three researchers [NE, IC and KL]. The analysts discussed their findings and, through discussion, they agreed upon the final set of labels. The interviews were analysed using the computer software program Atlas.ti (version 5.2; http://atlasti.com).

## Results

### Quantitative study

#### Participants

In total, 231 participants completed the survey. Approximately 950 email invitations were sent, resulting in a response rate of approximately 25%. The response rate varied between different professional groups. The most prevalent age group of participants was those aged between 51–60 years, 64% were male, 73% worked in an urban community setting, 50% had more than 20 years of work experience and more that 80% were currently providing services in the workers’ compensation setting at the time of the study. Participant characteristics are shown in Table 1.

**Table 1:** Participant characteristics (*n* = 231)^a^

Main category	Sub category	*N* (%)
Age	18–30 years	17 (7%)
	31–40 years	43 (19%)
	41–50 years	54 (23%)
	51–60 years	79 (34%)
	>60 years	37 (16%)
Sex	Female	83 (36%)
	Male	147 (64%)
Clinical specialty	Chiropractic	31 (13%)
	Clinical psychology	36 (16%)
	General practice	15 (6%)
	Injury management	14 (6%)
	Musculoskeletal & Occupational Medicine & Rheumatology	26 (11%)
	Pain & Rehabilitation Medicine	31 (13%)
	Physiotherapy	37 (16%)
	Surgery	39 (17%)
Work hours	Full time	58 (25%)
	Part time	172 (75%)
Clinical setting - geographical	Urban	169 (73%)
	Rural	36 (16%)
	Both	25 (11%)
Clinical setting - type	Public hospital	27 (12%)
	Private hospital	20 (9%)
	Community	96 (42%)
	Multiple settings	87 (38%)
Work experience	<10 years	41 (18%)
	10–20 years	73 (32%)
	>20 years	116 (50%)
Providing workers’ compensation services	No	39 (17%)
	Yes	191 (83%)

^a^231 participants were included, of which one participant did not complete the demographic characteristics

#### EBM perceptions across clinical specialties

On average, participants indicated that 76% of the treatments they recommend are evidence based (perceived EBM adherence). Regarding the EBM subscales, the median score for perceived EBM knowledge was Mdn = 4.0, the median for EBM attitudes was Mdn = 4.0, and the median for EBM behaviour was Mdn = 3.7 (on a scale from 1 to 5, in which 1 would imply strong barriers and 5 would mean no barriers). Wilcoxon comparisons showed that participants scored significantly lower on EBM behaviour than on EBM perceived knowledge (Z = −11.6, *p* < .001) and on EBM attitudes (Z = −11.8, *p* < .001). There were no differences between self-reported EBM knowledge and EBM attitudes (Z = −1.9, *p* = .062). Regarding the individual crude items, mean scores ranged from 3.3 on the item ‘In general, in my clinical field, payment systems can influence the decision about treatment’ to 4.4 on the item ‘I am aware of the evidence based practices in my field’ (on a scale from 1 = strongly disagree to 5 = strongly agree). Mean and median scores on the individual items are displayed in Table 2.

**Table 2:** Evidence Based Medicine perceptions

	Mean (SD)
1. EBM practice	
What percentage of the treatments you recommend and/or procedures you undertake is evidence based?	75.8 (20.0)
2. Knowledge	
a. I am *aware of* the evidence based practices in my field	4.4 (0.7)
b. I am *familiar with* the evidence based practices in my field	4.3 (0.6)
c. I have enough access to information about evidence based practices	4.0 (0.9)
d. I have/make time to keep myself up to date with evidence base practices	3.9 (0.9)
e. I am able to interpret the evidence base from the literature	4.1 (0.8)
Mean knowledge score:	4.2 (0.6)
3. Attitudes	
a. I feel confident that I can perform evidence based practice	4.2 (0.7)
b. I believe that evidence based practice leads to improved patient outcomes	4.1 (0.9)
c. I am motivated to adopt evidence based practice	4.2 (0.8)
Mean attitudes score:	4.2 (0.7)
4. Behaviour	
a. It is easy to apply evidence based treatment in my day to day practice	3.5 (1.0)
b. I am able to reconcile patient preferences with evidence based practice	3.7 (0.8)
c. There are enough resources/facilities (e.g. staff, educational material) to adhere to evidence based practice	3.4 (1.0)
d. I have enough time to apply evidence based treatment	3.7 (1.0)
e. My colleagues are supportive of the evidence base in my field	3.7 (1.0)
f. In general, in my clinical field, payment systems can influence the decisions about treatment	3.3 (1.2)
Mean behaviour score:	3.6 (0.6)

The scales for items 2a to 4f ranged from 1–5 (1 = strongly disagree, 5 = strongly agree)

#### EBM perceptions between clinical specialties

**Fig. 1:**
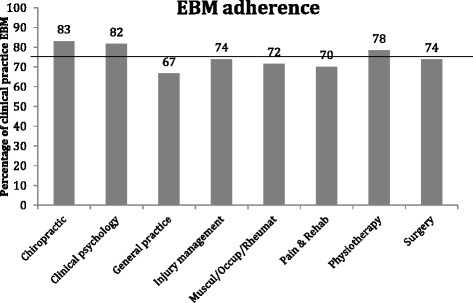
Percentage of clinical practice self-reported adherence to EBM, divided by clinical specialties. Horizontal line displays the average percentage across all clinical specialties (67%)

The percentage of evidence based treatments recommended between the different specialties ranged between 67% (reported by GPs) and 83% (reported by chiropractors) (Fig. 1). The main KW test showed significant differences between clinical specialties and self-reported EBM adherence (H (7) = 19.0, *p* = .008). Post hoc MW tests, using the general practitioner as a reference (being the lowest scoring specialty), revealed that the average (self-reported) percentage of evidence-based procedures by general practitioners was lower than the average (self-reported) percentage of evidence-based procedures by chiropractors (U = 137.0, *p* = .024) and psychologists (U = 381.5, *p* = .019). Nothwithstanding, it is important to stress that we assessed *self*-*reported* evidence based knowledge, not *actual* evidence based knowledge. General practitioners did not differ significantly in the (self-reported) amount of evidence-based procedures they recommend from injury management practitioners (U = 113, *p* = .747), musculoskeletal and occupational practitioners (U = 206, *p* = .779), pain and rehabilitation practitioners (U = 218.5, *p* = .740), physiotherapists (U = 334, *p* = .244), or surgeons (U = 335, *p* = .407).There were no differences between clinical specialties in answers to the question whether a payment system can influence decisions about treatment (H (7) = 10.3, *p* = .172). The mean score and standard deviations per clinical specialty to this item are shown in Table 3.

**Table 3:** Mean, Standard Deviation and Median per clinical specialty on item about payment system influencing decisions

In general in my field payment systems can influence decisions about treatment	Mean (SD)	Median
Chiropractic	3.1 (1.1)	3.0
Clinical psychology	3.4 (1.2)	4.0
General practise	3.4 (1.4)	4.0
Injury management	3.9 (1.0)	4.0
Musculoskeletal/Occupational Medicine/Rheumatology	3.0 (1.3)	3.0
Pain & Rehabilitation Medicine	3.7 (0.9)	4.0
Physiotherapy	3.4 (1.4)	3.0
Surgery	3.2 (1.2)	3.0

*SD* = Standard deviation

#### EBM comparisons between demographic and job characteristics

There were no differences between the demographic and employment characteristics regarding self-reported EBM adherence. The test statistics are reported in Table 4.

**Table 4:** Differences between demographic/job characteristics and EBM adherence

Demographic and job characteristic	Kruskal-Wallis H (df) or Mann–Whitney *U* test
Age (18–30/31–40/41–50/51–60/>60)	H (4) = 2.3, *p* = .686
Sex (Female/Male)	U = 5.6, *p* = .352
Years of work experience (<10/10–20/>20)	H (2) = 1.3, *p* = .510
Work hours (Full time/Part time)	U = 5.1, *p* = .746
Clinical setting (Urban/Rural/Both)	H (2) = 0.3, *p* = .867
Clinical setting (Public/Private/Community/Multiple)	H (3) = 0.9, *p* = .826
Providing services to workers’ compensation system (Yes/No)	U = 3.7, *p* = .986

### Qualitative study

#### Participants

In total, 15 participants consented to be interviewed (9 men and 6 women) from a total of 20 people approached. Five of the 15 were co-authors. All reported they provided services to the workers’ compensation system, ranging from 1 patient per week to 40 per week. The participant who reported seeing 40 patients per week was an occupational physician who only deals with workers’ compensation patients. Two participants had treated workers’ compensation patients in the past, but were not at the time of the interview. Participant characteristics are displayed in Table 5.

**Table 5:** Characteristics of interviewees (*n* = 15)

Characteristic	Subclass	Number
Sex	Female	6
	Male	9
Age group	30 to 39	3
	40 to 49	3
	50 to 59	6
	60 plus	3
Work hours (clinical work)	Part-time	6
	Full-time	9
Work experience in clinical practice	<10 years	3
	10–20 years	4
	>20 years	8
Professional background	Physiotherapy	5
	Psychology	2
	General practice	1
	Injury management	1
	Occupational medicine	1
	Pain & Rehab	3
	Surgery	2
Clinical setting – geographical	Urban	11
	Rural	1
	Both	3
Clinical setting – type	Public	3
	Private	6
	Public & Private	4
	Community	2

#### Opinions about an EBM tool in the workers’ compensation setting

Some of the opinions about the tool were positive, for instance, stating that the tool could improve trust and provide greater guidance for clinicians, it could reduce over-servicing and it could assist in managing patient expectations. The majority of opinions, however, included concerns that the tool would not take into account clinical judgement; the quality of the evidence may be poor and thus result in potentially poor decisions, and that there would be insufficient evidence for decisions. Additional concerns included: a lack of risk assessment tools, the likelihood of contentious critical appraisal and that claims managers usually have no clinical training. Themes are described in more detail below. A summary is provided in Table 6.

**Table 6:** Summary of findings about EBM tool in the workers’ compensation setting

Issue	Comment
Trust and guidance for clinicians	Automatically approval of treatment could be perceived as a sign that that the claims managers trust the clinicians’ judgement
Limiting over-servicing	Recommendations about MRIs and certain surgeries could limit over-servicing, although solicitors could still get it anyway
Patient expectations and claimant monitoring	Tool may assist with managing patient expectations, as it sets timeframes about RTW
Individual differences & psychosocial factors	Tool may inadequately consider individual differences and psychosocial factors. Most patients are not one size fits all
Clinical judgement	Clinical judgement is important. Tool should not overpass clinical judgement
Patient preference	Some patients prefer to have non evidence based treatments but in general those patients can be convinced to value EBM
Quality of evidence	For many topics the evidence is not replicated, or very specific to certain populations
Quantity of evidence	Lack of evidence should not imply denial of treatment
Timeliness and risk assessment	Tool may not adequately assess risk of prolonged recovery, and therefore not sufficiently focus on timely treatment
Critical appraisal and guideline development	Interpretation of evidence is dependent on who does the interpretation. American tool might not be applicable in Australia
Claims managers using the EBM tool	Inexperience or limited training for claims managers could lead to rigid usage and unfair denials
Tool is no solution	Tool may not assist with the 20% most problematic cases, and does not recognise employer factors that prolong return to work

##### Trust and guidance for clinicians

Some interviewees were of the opinion that the automatic approval of treatment would improve the clinicians’ trust in the process. Another participant stated that a potentially positive characteristic of the tool was that it is the first that provides an indication of the number of treatment sessions the patient has received with comparison to optimal number of sessions supported by evidence. Further, they believed that by indicating how many treatment sessions on average are effective for a typical patient, the tool would provide an opportunity to monitor, detect and prevent chronic disability.

##### Limiting over-servicing

Some participants acknowledged that an EBM tool might limit over-servicing or inappropriate servicing, for example, with respect to MRI scans, or knee arthroscopy for degenerative meniscal tears. On the other hand, some interviewees doubted whether the tool would be able to prevent over-servicing, because solicitors can still inform their client (the patient) to go ahead and seek services anyway, and claim the cost back. Another participant thought the tool may not necessarily prevent non-recommended treatment because health care practitioners do not tend to go into detail about the management plan, which makes it difficult for the claims manager to detect whether it involves unnecessary treatment.

##### Patient expectations and claimant monitoring

Some participants thought the tool could assist with managing patient expectations about return to work. For example, if the health care professional communicated return to work timeframes to the patient, the patients will have more informed expectations about return to work and whether they are ahead or behind schedule. Another participant suggested the tool could also (positively) alter the claims manager’s attitude, given the tool would enable the manager to compare the return to work days of the injured claimant to the average claimant. It was thought this would improve the claims managers’ awareness of whether the claimant is on track and be more solution-focused about (barriers to) return to work, rather than just focusing on closing the claim.

##### Individual differences and psychosocial factors

Interviewees raised concerns as to whether the tool would take into account individual differences and psychosocial factors. They stated that EBM is usually based on averages and selective populations, whereas most patients have individual needs and are therefore not “one size fits all”. Interviewees also emphasised that psychosocial factors, such as mood, work satisfaction and whether they feel that their employer is supporting them, are known to be superior predictors of the outcome than the nature of the injury itself. They were also concerned that the tool would not sufficiently take into account the type of work and the duties performed.

##### Clinical judgement

Some interviewees were concerned that the tool would overrule clinical judgement. They stressed that there is value in expert clinical opinions, based on years of experience. It was emphasised that clinical experience is needed to use and interpret the psychosocial assessments. It was emphasised that patients are best assessed by a health professional who has assessed the patient and is aware of their needs. It was perceived to be undesirable and even threatening that a case manager might dictate the treatment. They were concerned that the tool was going to be “recipe” driven and too prescriptive: “Where is it going to end, technicians doing our job”.

##### Patient preferences

Interviewees did not seem concerned that the tool may conflict with patients sometimes having different preferences than EBM recommendations. When asked about non-EBM preferences, participants said, for example, that some patients may request massage rather than receiving active physiotherapy, or some patients may prefer psychodynamic psychotherapy over the recommended cognitive behavioural therapy (CBT). In the case where a patient requests non-evidence based treatment (that is, treatments without evidence of benefit or conflicting evidence), several interviewees felt confident they could explain that such treatment may not be effective and that an evidence-based treatment is recommended. The participating surgeons reported that patients usually follow the surgeon’s advice, however, some patients are also reluctant to have surgery, even if advised by the surgeon.

##### Quality of evidence

Some participants were concerned that the tool, or the people applying the tool, would not take sufficiently into account the quality of the evidence. They questioned whether the tool could evaluate studies that are possibly flawed in their methodology and conclusions. Participants also raised the debatable issue of what defines a good quality outcome is and for whom. For instance, a good outcome for the patient may not be a good outcome for the insurance company or the treatment provider. Furthermore, some treatments might result in positive health outcomes for patients, but if only 1 out of 100 go back to work, the insurer may not approve the program. Participants were also concerned whether the tool would recommend appropriate assessments. They reported that the tool should not recommend physical assessments, but also recommend psychosocial assessments, such as for the patients’ perception related to the severity of their pain and its effects.

##### Quantity of evidence

Interviewees were concerned that treatments, for which there is a lack of evidence, would be denied by insurance companies. They emphasised that the fact that a treatment is not currently supported by research evidence does not mean that it does not work. Chronic pain and rehabilitation programs were considered to have a poor evidence base, and some types of treatments are more likely to be investigated than others. For example, a psychologist said there is a strong interest for therapies that are manualised and that can be delivered quickest, such as CBT, but that implies that other potentially beneficial therapies are less likely to be investigated and thus approved.

##### Timeliness and risk assessment

A number of participants mentioned that there is more to treatment approval than providing recommendations about evidence based treatment. They were concerned that the tool did not apply early risk assessment in order to be able to facilitate early interventions. They considered it a shortcoming that claims managers currently do not acknowledge or implement risk assessment tools, which identify workers at risk of limited or delayed recovery. Participants strongly emphasised that some health professionals should be getting referrals earlier, not just referrals after everything else has been tried, particularly in relation to pain management.

##### Critical appraisal and applicability

There were some concerns that the process of interpreting, synthesising, and developing recommendations is potentially dependent on the personalities of those appraising the evidence. Also it was believed that cultural factors may influence the interpretation of evidence and therefore this was considered to be an important consideration for the tool. Even though the recommendations are based on international evidence, it was perceived that a North American advisory board might favour American research or be less tolerant of certain types of treatment. Participants also commented that the USA has a different health care system and a more litigious society compared to Australia, and that this may result in recommendations that are based on such a health system and which may not be applicable to the Australian scene.

##### Claims managers using the EBM tool

There were concerns about how claims managers would apply the tool. Claims officers are often considered fairly junior, non-health or clinically trained, “looking for a tick box sort of modality”. Several interviewees noted the stressful work environment of claims managers, the high case load and a high turnover, all considered part of the problems in workers’ compensation in general. Interviewees were worried claims managers would misuse medical evidence to deny treatment, or stop paying for treatment if the patient is not back at work by a particular time. They were worried insurers will use the tool in a punitive manner, as a “big stick”.

##### Tool is no solution

Several participants questioned whether this tool would be a solution for the treatment approval process. They thought it might streamline the initial treatment approval process for those cases that were non-problematic already, but the 10–20% of difficult cases could remain disputed. In addition, it was reported that, if the workplace is not supportive or facilitating a RTW, then even though treatment is effective, the worker is unlikely to go back to work. Overall interviewees showed some lack of trust in the workers’ compensation process, and some interviewees were critical of the system, for example: “There is a fatigue factor in being micromanaged by claims managers who are children in the industry”.

## Discussion

This study investigated health practitioners’ perceived knowledge, attitudes and behaviours with reference to 1) evidence-based medicine in general, which was investigated by a quantitative study, and 2) an EBM tool in the workers’ compensation setting specifically, which was investigated by a qualitative study. The data collected from the health professional sample involved in the quantitative study revealed that NSW health care practitioners believe 76% of their clinical practice is evidence based. This percentage is comparable to a recent study among Australian physiotherapists and chiropractors showing that, after an intervention, 79% of participants were compliant with clinical whiplash guidelines (whereas before the intervention compliance was 58%) [11]. On the other hand, 76% is high compared to a study among Australian general practitioners, showing that only about 20% of low back pain patients received the care that is recommended by low back pain guidelines [12]. Our study investigated EBM practices in general, rather than being related to a specific condition, and the percentage of evidence based treatment was self-reported, so it is not possible to draw objective conclusions about the finding.

The study found differences between clinical specialties and EBM behaviour. General practitioners perceived more barriers than other clinical practitioners. This difference may be partially due to different treatment contexts. For example, general practitioners are more aware of the injured worker in their wider context, meaning their family, past health and coping styles. They might therefore perceive more barriers around applying EBM due to the concern that an EBM (tool) does not take into account individual patient differences and complex psychosocial factors. On the other hand, psychologists also treat injured persons within a wider context, but they scored highly on self-reported EBM adherence. General practitioners also deal more with a wider spectrum of diseases, so perhaps it is more difficult to keep abreast of with guidelines across all conditions [13]. Finally, general practitioners have a gatekeeper role in the compensation system, which can create significant pressure for the professional involved [14].

In the qualitative study, the themes identified about the EBM tool being applied in the workers’ compensation setting supported a range of views that have been published about evidence based health care, such as the importance of patient preferences and clinical judgement, influential critical appraisal of the evidence, lack of quality evidence, and the lack of evidence for the majority of care [4, 15–18]. Interviewees particularly emphasised the importance of clinical judgement when applying the evidence to individual patients, reflecting Sackett’s original and widely accepted definition of evidence based medicine [19]. Notably, participants in the qualitative study seemed much more critical of an EBM tool applied in the workers’ compensation setting than the participants in the quantitative study. One explanation maybe that many clinicians will feel threatened by such a tool, given EBM decisions are being made by an external party (the claims managers). In addition to claims managers being an external party, clinicians regard their decisions on treatment with scepticism since claims managers are often junior without a health training background.

Another important theme that was mentioned in relation to the EBM tool applied to the workers’ compensation setting, was the lack of screening tools and lack of acknowledgement of the influence of environmental and psychosocial factors, such as work dissatisfaction, family dynamic problems at home and coexisting illness in older workers. Psychosocial factors are important determinants of outcome after work injury [20, 21]. The biopsychosocial approach has been widely advocated in medicine, but, based on communication with insurance companies, the biopsychosocial model does not seem to have been fully adopted into the compensation systems as yet. As far as we know, there is no general insurance policy for screening for the influence of psychosocial factors. Anecdotal evidence suggests that claims managers may be hesitant to provide early treatment due to the belief that this would increase the costs. However, studies in a workers’ compensation setting have shown that early screening and intervention in people with musculoskeletal injuries with high chances of poor recovery resulted in significant cost *reduction* [22, 23]. This approach has also been shown to result in improved outcomes and reduced cost in low back pain in the UK [24], and is now being investigated in Australia for the management of whiplash [25]. For an optimal treatment approval process, it is recommended that claims managers in the compensation system not only approve evidence based treatment, but also encourage the use of psychosocial assessment tools, that for example, predict chronic pain and disability, after which they should offer the earliest intervention available.

A meta theme that was developed from the interviews, that could explain some of the concerns about the EBM tool, may be the significant negative experiences some health professionals have had with the workers’ compensation system. Several interviewees were sceptical of an EBM tool, reporting that similar initiatives to improve the scheme had been undertaken in the past and these did not succeed (either). A recent paper about the scheme argued that ‘in spite of an abundance of government recommendations and scholarly evidence prioritising timely return to work for injured workers, the NSW Workers’ Compensation Scheme systematically fails to support this objective’ [26]. The interaction between HCP and the claims managers in the workers’ compensation scheme has been found challenging [27]. It seems that, besides or before implementing an EBM tool, the interaction between HCP and claims managers, and claims manager training should be improved. Early case conferencing, in which HCP, claims manager and patient are sitting in the room to discuss realistic goals and concerns, may lead to better understanding of the complexities associated with workplace injuries. Preferably, the workplace needs to be involved too [28]. This study focused on the interaction between HCP and the compensation system only, but it should be emphasised that the employer also plays an important role in the success of treatment and the return to work process. In order to achieve an effective and sustainable compensation system, all stakeholders should be involved [15].

### Strength and limitations

A strength of the study was the moderately large sample of practitioners surveyed, providing sufficient statistical power to determine differences. Participants had substantial clinical experience, including experience within the workers’ compensation system. Potential limitations included the relatively small number of responses from some professional groups. Findings, especially in relation to general practitioners, should be interpreted with caution. While effort was made to ensure representative opinions, the study could have been limited with respect to generalisability of the clinical groups surveyed. Furthermore, the questionnaire was self-report based and self-reporting of EBM practise (or knowledge thereof) does not necessarily mean clinicians are actually practising EBM. Also, the possibility of selection bias of the participating practitioners is acknowledged and this may have resulted in respondents having a greater knowledge and use of EBM than practitioners generally.

## Conclusions

Overall, it is concluded that HCP in NSW, Australia, were supportive of EBM, however, many had concerns about the implementation in clinical practice, when operating in workers’ compensation settings. It is concluded that EBM should be applied in a flexible manner, taking substantial account of the clinical expertise and judgement of the practitioners, patient differences and psychosocial contexts. If an EBM tool is going to be implemented, adequate training of claims managers is recommended as well as an introduction to the tool for clinicians. It is also concluded that special attention should be given to general medical practitioners before an EBM tool is implemented. Lastly, it is recognized that the treatment approval process in the Workers’ Compensation system in NSW is a complex and sensitive process, which could be improved if interactions between claims managers and HCP were enhanced. It is important that careful, well-informed decisions are made about treatments for those people with injuries proceeding through the system.

## Additional file

Additional file 1: Questionnaire EBM. (DOCX 18.5 kb)

## Abbreviations

EBM: Evidence based medicine
HCP: Health care professionals
NSW: New South Wales
