# Supplementary material for: Health care professionals’ attitudes towards evidence-based medicine in the workers’ compensation setting: a cohort study
Source: BMC Med Inform Decis Mak. 2017 May 22;17:64. doi: 10.1186/s12911-017-0460-2 (PMC5440905; doi:10.1186/s12911-017-0460-2)
Supplement: Additional file 1: — Questionnaire EBM. (DOCX 18.5 kb) [file 12911_2017_460_MOESM1_ESM.docx]

**Additional file – Questionnaire EBM**

Thank you for participating in a short questionnaire investigating your attitudes towards evidence based medicine.

The questionnaire will consist of statements about evidence based medicine, followed by some questions about demographic and job characteristics.

The questionnaire is anonymous.

**Evidence Based Medicine**

In health care, there is often a **gap between evidence and practice**. A Lancet publication in 2003 reported that about 30–40% of patients do not receive care according to present scientific evidence (Grol & Grimshaw, 2003). In 2015, a report by the Grattan institute stated that far too many people get a treatment they should not get, even when the evidence is clear that it is unnecessary or doesn’t work (Duckett & Breadon, 2015).

A simple example of the gap between evidence and practice is hand hygiene in hospitals. The evidence says that hand hygiene among personnel could prevent about 15% to 30% of the hospital acquired infections. Nevertheless, in practice, hand hygiene compliance is generally less than 50% (Huis et al, 2012).

Health care professionals report various barriers to applying evidence based medicine in their practice. Some say they are not fully aware of the current evidence, others feel it is difficult to reconcile evidence based medicine with patient preferences, others say they don’t have enough time to apply evidence based medicine, or that it is financially advantageous to apply *non*-evidence based practices.

In this questionnaire, we would like to investigate whether you perceive any barriers to applying evidence based **practices** and evidence based **guidelines.** We also want to investigate whether you use an evidence based guideline **tool** to get better access to evidence based medicine.

**Practices**

The first questions are about adopting evidence based practices. Applying evidence based practices means that you use peer reviewed publications, or other peer reviewed materials, that provide evidence for the effectiveness for specific treatments.

1. **On estimation, what percentage of the treatments you recommend and/or procedures you undertake is evidence based? (0-100%)**

…..

1. **Knowledge**

**To what extent do you agree with the following statements about your knowledge of evidence based practices?**

|  | strongly disagree | disagree | neutral | agree | strongly agree |
| --- | --- | --- | --- | --- | --- |
| - 1. I am *aware of* the evidence based practices in my field |  |  |  |  |  |
| - 1. I am *familiar with* the evidence based practices in my field |  |  |  |  |  |
| - 1. I have enough access to information about evidence based practices |  |  |  |  |  |
| - 1. I have/make time to keep myself up to date with evidence base practices |  |  |  |  |  |
| - 1. I am able to interpret the evidence base from the literature |  |  |  |  |  |

1. **Attitudes**

**To what extent do you agree with the following statements about your attitudes towards evidence based practices?**

|  | strongly disagree | disagree | neutral | agree | strongly agree |
| --- | --- | --- | --- | --- | --- |
| - 1. I feel confident that I can perform evidence based practice |  |  |  |  |  |
| - 1. I believe that evidence based practice leads to improved patient outcomes |  |  |  |  |  |
| - 1. I am motivated to adopt evidence based practice |  |  |  |  |  |

1. **Behaviour**

**To what extent do you agree with the following statements about your behaviour towards evidence based practices?**

|  | strongly disagree | disagree | neutral | agree | strongly agree |
| --- | --- | --- | --- | --- | --- |
| - 1. It is easy to apply evidence based treatment in my day to day practice |  |  |  |  |  |
| - 1. I am able to reconcile patient preferences with evidence based practice |  |  |  |  |  |
| - 1. There are enough resources/facilities (e.g. staff, educational material) to adhere to evidence based practice |  |  |  |  |  |
| - 1. I have enough time to apply evidence based treatment |  |  |  |  |  |
| - 1. My colleagues are supportive of the evidence base in my field |  |  |  |  |  |
| - 1. In general, in my clinical field, payment systems can influence the decisions about treatment |  |  |  |  |  |

**Guidelines**

The next questions are about adopting evidence based guidelines. Using evidence based guidelines means you use publications that summarise the evidence related to multiple aspects of a health condition and suggest treatments that make up multiple components of a management plan. Examples of evidence based guideline are the NSW Whiplash Guideline and the Australia and New Zealand Guideline for Hip Fracture Care.

1. **To what extent are you adhering to evidence based *guidelines*?**

|  | strongly disagree | disagree | neutral | agree | strongly agree |
| --- | --- | --- | --- | --- | --- |
| - 1. I am aware of evidence based guidelines available for my speciality |  |  |  |  |  |
| - 1. I believe evidence based medicine guidelines are useful in my field |  |  |  |  |  |
| - 1. I usually use at least one evidence based guideline in my practice |  |  |  |  |  |

**Tool**

The next question is about whether you use an electronic evidence based guideline tool. An electronic evidence based guideline tool gives you an extensive overview of evidence based treatments and guidelines for a condition and provides recommendations based on the quality of evidence. Examples of evidence based guideline tools are the ODG (Official Disability Guidelines), or MD Guidelines.

**Do you use an electronic evidence based guideline tool?**

- No
- Yes, I use the following tool: ……

**Keywords**

1. **What positive keywords do you associate with Evidence Based Medicine?**

…………..

1. **What negative keywords do you associate with Evidence Based Medicine?**

………….

-------------------------------------------< page break >---------------------------------------------------

**Demographic and Job characteristics (page 2 out of 2)**

The final questions are about your age, gender and clinical experience.

1. What is your age?

- 18-30
- 31-40
- 41-50
- 51-60
- > 60

1. What is your gender?

- Female
- Male

1. What is your clinical specialty?

- chiropractic
- clinical psychology
- general practice
- injury management
- musculoskeletal medicine
- occupational medicine
- pain medicine
- physiotherapy
- rehabilitation medicine
- rheumatology
- surgery, hand
- surgery, orthopaedic
- surgery, other
- other …….

1. What is the number of years of work experience in your current field of practice?

- < 10 years
- 10 - 20 years
- > 20 years

1. Do you work part time or full time?

- Part time
- Full time

1. Do you work in an urban or rural clinic?

- Urban
- Rural
- Both

1. In what clinical setting do you work?

- Public hospital
- Private hospital
- Community
- Multiple settings

1. In the last 12 months, did you provide services to the workers’ compensation scheme?

- Yes
- No

This is the end of the questionnaire.

**Thank you very much for participating!**
